# Supplementary material for: Exposure to pollutants for household cooking and lighting and pediatric post-discharge mortality following a severe infection in Uganda
Source: PLoS One. 2025 Jul 9;20(7):e0326105. doi: 10.1371/journal.pone.0326105 (PMC12240310; doi:10.1371/journal.pone.0326105)
Supplement: S1 Table — (DOCX) [file pone.0326105.s001.docx]

**S1 Table. Distribution of proxy exposure variable by type of cooking fuel, cooking location, primary source of household lighting, and mortality.**

| Exposure Level | Type of Cooking Fuel | Cooking Location | Primary Source of Household Lighting | Alive | Dead (%) |
| --- | --- | --- | --- | --- | --- |
| Dual | Pollutant | Indoors | Pollutant | 1017 | 90 (8.1%) |
| Single | --- | --- | Pollutant | 357 | 28 (7.3%) |
| Single | Pollutant | Indoors | --- | 3752 | 245 (6.1%) |
| Minimal | --- | --- | --- | 1390 | 76 (5.2%) |

*Note*. The proxy exposure variable was stratified into three exposure levels: dual exposure to both pollutant cooking fuel sources used indoors and pollutant household light sources; single exposure to pollutant fuel sources used for household light OR cooking indoors; and exposure to minimal pollutant fuel sources for cooking and household lighting.
