# Supplementary material for: Exposure to pollutants for household cooking and lighting and pediatric post-discharge mortality following a severe infection in Uganda
Source: PLoS One. 2025 Jul 9;20(7):e0326105. doi: 10.1371/journal.pone.0326105 (PMC12240310; doi:10.1371/journal.pone.0326105)
Supplement: S2 Table — (DOCX) [file pone.0326105.s002.docx]

**S2 Table**. **Stratified analysis categorized by discharge diagnosis exploring adjusted risk ratios comparing the risk of death within 6-months post-discharge for individuals with dual or single exposure to pollutant fuel sources for cooking and household lighting compared to minimal.**

| Discharge Diagnosis | Adjusted Risk Ratio^1^ (95% CI) |
| --- | --- |
| Malaria |  |
| Dual exposure^2^ | 1.74 (0.76, 4.50) |
| Single exposure^2^ | 1.33 (0.62, 3.28) |
| Pneumonia |  |
| Dual exposure^2^ | 0.78 (0.44, 1.34) |
| Single exposure^2^ | 1.09 (0.74, 1.65) |
| Malnutrition |  |
| Dual exposure^2^ | 0.73 (0.37, 1.42) |
| Single exposure^2^ | 0.66 (0.38, 1.20) |
| Anemia |  |
| Dual exposure^2^ | 1.26 (0.78, 2.05) |
| Single exposure^2^ | 1.12 (0.75, 1.74) |
| Respiratory Illness |  |
| Dual exposure^2^ | 0.94 (0.56, 1.56) |
| Single exposure^2^ | 1.11 (0.76, 1.66) |

*Abbreviations*: CI, confidence interval

^1^The multivariable model adjusts for age, sex, distance of home to facility, maternal education, and maternal HIV status.

^2^Reference group: Individuals categorized as having minimal exposure to pollutant fuel sources for cooking and household lighting.
