# Supplementary material for: Exposure to pollutants for household cooking and lighting and pediatric post-discharge mortality following a severe infection in Uganda
Source: PLoS One. 2025 Jul 9;20(7):e0326105. doi: 10.1371/journal.pone.0326105 (PMC12240310; doi:10.1371/journal.pone.0326105)
Supplement: S3 Table — (DOCX) [file pone.0326105.s003.docx]

**S3 Table. Full results of the bivariate Poisson regression investigating the relationship between 6-month post discharge mortality with dual or single exposure to pollutant fuel sources for cooking and household lighting compared to minimal.**

| Explanatory Variables | Regression Coefficients | Standard Error | P value | 95% Confidence Intervals |
| --- | --- | --- | --- | --- |
| Intercept | -2.96 | 0.11 | <0.0001 | -3.18, -2.74 |
| Exposure |  |  |  |  |
| Dual | 0.45 | 0.15 | 0.01 | 0.15, 0.75 |
| Single | 0.18 | 0.13 | 0.15 | -0.06, 0.43 |
