# Supplementary material for: Exposure to pollutants for household cooking and lighting and pediatric post-discharge mortality following a severe infection in Uganda
Source: PLoS One. 2025 Jul 9;20(7):e0326105. doi: 10.1371/journal.pone.0326105 (PMC12240310; doi:10.1371/journal.pone.0326105)
Supplement: S4 Table — (DOCX) [file pone.0326105.s004.docx]

**S4 Table. Full results of the multivariate Poisson regression investigating the relationship between 6-month post discharge mortality with dual or single exposure to pollutant fuel sources for cooking and household lighting compared to minimal, adjusted for age, sex, distance of home to facility, maternal education, and maternal HIV status.**

| Explanatory Variables | Regression Coefficients | Standard Error | P value | 95% Confidence Intervals |
| --- | --- | --- | --- | --- |
| Intercept | -2.47 | 0.19 | <0.0001 | -2.84, -2.10 |
| Exposure |  |  |  |  |
| Dual | 0.26 | 0.15 | 0.09 | -0.04, 0.56 |
| Single | 0.08 | 0.13 | 0.54 | -0.17, 0.33 |
| Age | -0.01 | 0.01 | 0.01 | -0.02, -0.01 |
| Sex |  |  |  |  |
| Male | 0.07 | 0.09 | 0.48 | -0.12, 0.25 |
| Distance to home to facility | 0.01 | 0.01 | <0.0001 | -0.01, 0.01 |
| Maternal education |  |  |  |  |
| No school | -0.30 | 0.25 | 0.22 | -0.79, 0.18 |
| P4 to P7 | -0.42 | 0.15 | 0.01 | -0.72, -0.13 |
| S1 to S6 | -0.69 | 0.16 | <0.0001 | -1.01, -0.37 |
| Post secondary | -1.06 | 0.23 | <0.0001 | -1.52, -0.60 |
| Don’t know | 0.22 | 0.38 | 0.56 | -0.53, 0.97 |
| Maternal HIV Status |  |  |  |  |
| Positive | 0.26 | 0.15 | 0.08 | -0.04, 0.56 |
| Unknown | 0.21 | 0.21 | 0.32 | -0.21, 0.63 |
