# Supplementary material for: Exposure to pollutants for household cooking and lighting and pediatric post-discharge mortality following a severe infection in Uganda
Source: PLoS One. 2025 Jul 9;20(7):e0326105. doi: 10.1371/journal.pone.0326105 (PMC12240310; doi:10.1371/journal.pone.0326105)
Supplement: Attachment — Submitted filename: Response to Editor.docx [file pone.0326105.s008.docx]

Hello Dr. Srijan Lal Shrestha,

Thank you for your thorough review of our manuscript and the constructive comments that you provided. We appreciate the time and effort you have invested in assessing our work.

Our team has addressed all comments and additional journal requirements detailed in the decision email. We have provided our responses to all major/minor comments below in an orderly fashion. We have also ensured that our manuscript meets PLOS ONE’s style requirements and uploaded a complete copy of PLOS’ questionnaire on inclusivity in global research. Regarding data sharing, due to the sensitive nature of the patient data and the risk for re-identification, data is only available through reasonable request to the Pediatric Sepsis Data CoLaboratory. All data and collection tools are available through the Smart Discharges Dataverse. The dataset is published and can be viewed using this link:  <https://doi.org/10.5683/SP3/ZLZQOG> (Citation: Dhutt GS, Zhang C, Kumbakumba E, Tagoola A, Moschovis P, Businge S, et al. Exposure to pollutants for household cooking and lighting and pediatric post-discharge mortality following a severe infection in Uganda [Internet]. V1 ed. Borealis; 2025. Available from: <https://doi.org/10.5683/SP3/ZLZQOG> ). Access to the data can be requested through the link above. Access to data is granted on a case-by-case basis following approval from the authors and the Data Governance Committees.

Note: page and line numbers are provided for new text which have been added to the manuscript. The page and line numbers correspond to those in the manuscript document with tracked changes titled “Revised Manuscript with Track Changes”.

We hope our revisions meet your expectations, and we are happy to address any further concerns. Thank you for considering our manuscript for publication.

**Major comments**

**1. Even though the research was conducted to establish relationship between HAP exposure and child mortality, it showed otherwise. In this context, it is very essential to discuss about the importance about the study. Authors have mentioned lots of the limitations of the study. It also requires to justify the benefits of the study. Consequently, why is it important to publish the findings of the study in the presence of so many limitations in the study? This needs to be clarified and discussed.**

Thank you for this comment. We agree that our work showed otherwise however, our work uses a similar methodology for exposure data as many other research studies that show both similar and divergent results (see lines 287-299). This underscores our argument that using proxy variables may not be reliable and accurate. Regardless, our study is of general relevance to the research community in that it examines an area not previously explored, namely the impact of HAP on short term outcomes in the context of illness recovery and convalescence. Our research team focuses primarily on post-discharge recovery, recognizing that as many children die following discharge as during the acute phase of illness. The impact of HAP on this phase of recovery has not previously been explored, and regardless of the typical limitations to HAP exposure measurement that may exist in our analysis, these results must spur further investigation into the increasingly topical and relevant area of the post-acute illness period. It is this distinction makes our study unique and relevant.

We have included the following in our discussion:

*Page #21, line 316-321 “However, recognizing that as many children die following discharge as during the acute phase of illness, the impact of HAP on this phase of recovery has not previously been explored, and regardless of the typical limitations to HAP exposure measurement that may exist in our analysis, these results must spur further investigations into the increasingly topical and relevant area of the post-acute illness period. It is this distinction which makes our study unique and relevant.*”

**2. Introduction part is very short for a research paper which has such an important issue. Authors should include important and related literature reviews which have quantification of effects of HAP on childhood mortality.**

Thank you for your suggestion. We have included the following in the Introduction:

*Page #4, line 80-83* “*This practice results in harmful indoor air pollution and, in 2020, was reported to be associated with about 3.2 million deaths, disproportionately impacting children in Sub-Saharan Africa due to increased pollution levels resulting from rapid urbanization and industrialization in this region. (1-4)”*

*And on Page #5, line 88-93 “Children further face increased risk due to extended exposure to particulate matter from household air pollution from engagement in household activities such as cooking and household lighting. (1, 6) In the Sub-Saharan African context, the use of household pollutant fuel sources (e.g., kerosene or coal) has been shown to increase the risk of all-cause child mortality, with current estimates being as high as a 126% increase in mortality due to pollutant cooking fuel source use inside the household. (8-14)”*

**3. Why this research based upon only 6 hospital patients is representative of Uganda? How many critical child caring hospitals are there in Uganda? This is a critical question which needs to be justified. Otherwise, mentioning of Uganda will not be accepted. Instead, authors should mention the representation of results for only the selected sub-groups or regions, if this is the case.**

We agree that not all hospitals in Uganda were used in the study. However, we feel that the hospitals are representative of Uganda in that the public and faith-based facilities included in this study consist of a catchment area comprising 30 districts with an approximate population of 8.2 million individuals, of which 1.4 million were children younger than 5 years at the time data collection was conducted. (See Uganda Bureau of Statistics. Population and censuses. 2022. https://www.ubos.org/explore-statistics/20/). Hence, we believe the 6 hospitals chosen are representative of Uganda as these hospitals represent the major facilities caring for critically ill children within the regions covered (Central Uganda, Southwest Uganda, Eastern Uganda). Having said this, we are happy to remove the mention of Uganda if the editor feels it is not sufficiently justified.

**4. Authors have used logistic regression for analysis by specifying only the adjusted odds ratios. Instead, it is suggested to specify the full logistic regression model with all the explanatory variables included along with their estimates and relevant statistics. Also, how were the interaction effects assessed? It needs to be clarified.**

Based on the feedback in the minor revisions section, we have reanalyzed our data using Poisson regression with robust standard errors and provided risk ratios as opposed to odds rations. The full Poisson regression model with all the explanatory variables and their estimates/relevant statistics are provided in the supplementary file titled “S3_Table; S4_Table”. The results section of the manuscript provides the unadjusted and adjusted risk ratios in Table 3, with a mention of all the variables that were included in the adjusted model.

We have added the following to the methods section regarding how the interaction effects were assessed:

*Page 9, lines 180-182: “To test for interaction, models with and without the interaction term were compared using the likelihood ratio test. A p-value of < 0.05 was used to determine whether interaction terms were statistically significant.”*

**5. Assumptions and model adequacy tests required for the logistic model have not been mentioned in the paper. This is also essential in modeling perspective.**

We have reanalyzed our data using Poisson regression with robust standard errors and provided risk ratios instead of odds ratios.

We have added the following to the methods section regarding how assumptions of the model were checked:

*Page 9, lines 174-176: “The analysis consisted of using Poisson models with robust standard errors. We checked for outliers, multicollinearity between variables, and plotted residuals to check for independence.”*

**Minor comments**

**1. It is better to mention about inclusion and exclusion criteria of the research. If mentioned, it should be better in this sub-heading.**

We have changed the “Study population” heading to “Study population inclusion and exclusion criteria” to reflect this.

**2. Why 2 citations of data collection method? Only the citation whose data collection method matches exactly with the research needs to be mentioned.**

We have removed the second citation and kept the citation whose data collection method matches exactly with the research.

**3. Elaborate on statement that the analysis did not adjust for multiple comparisons.**

This statement has been removed from the manuscript.

**4. Role of funding source/ ethical clearance need not be mentioned in the main text of a paper. They will be mentioned as per the journal guidelines.**

We have removed this section from the main text of the paper.

**5. Table 1: It is also required to mention about the mortality status along with exposure and other variables.**

We have added mortality status to Table 1.

**6. Why relative risks (RRs) are not reported for a prospective cohort study results?**

We have replaced the odds ratios with risk ratios in the manuscript.
