# Supplementary material for: Exposure to pollutants for household cooking and lighting and pediatric post-discharge mortality following a severe infection in Uganda
Source: PLoS One. 2025 Jul 9;20(7):e0326105. doi: 10.1371/journal.pone.0326105 (PMC12240310; doi:10.1371/journal.pone.0326105)
Supplement: Attachment — Submitted filename: Response to Reviewers.docx [file pone.0326105.s009.docx]

Hello Dr. Srijan Lal Shrestha,

Thank you to you and the reviewers for your comments and feedback. As always, we appreciate the time and effort you have invested in assessing our work.

We have reviewed our reference list for completeness and correctness. To the best of our knowledge, there are no references cited which have been retracted.

Please find below our responses to the comments provided.

Note: page and line numbers are provided for new text which have been added to the manuscript. The page and line numbers correspond to those in the manuscript document with tracked changes titled “Revised Manuscript with Track Changes”.

We hope our revisions meet your expectations, and we are happy to address any further concerns. Thank you for considering our manuscript for publication.

**Reviewer #1 Comment:**

**Authors have addressed the comments provided by the previous reviewer. Since the result did not showed relationship with HAP exposure and child mortality which is against established evidence from many studied. Therefore, further discussion on contextual factors and reasons is desirable.**

Thank you for this comment. We completely agree and recognize that it is important to situate our findings based on contextual factors in the hopes that it spurs future research to further explore our area of interest. While we agree that a significant body of evidence has linked HAP with childhood mortality, very little work has been done among children at high risk of mortality over the short term (i.e. 6 months post-discharge as in this study). We also agree that objective measures of PM2.5 exposure would have added a further element of robustness to our analysis and we have included this limitation in our discussion. We believe that this research justifies the exploration of long-term impacts of HAP on mortality following acute illness to determine if, over time, the effects of air pollution are additive.

Our limitations section already outlines exposure measurement and confounding as two key contextual factors relevant to our results. To add additional clarity, we have added another sentence to the limitations which states that:

*Page #21, line 313-315 “Future studies should incorporate more objective measures of HAP, such as devices which directly measure PM2.5 exposure, to more accurately measure the relationship between HAP and post-discharge outcomes.*

With regards to deviations from existing literature, we have emphasized the unique nature of this study which makes it novel. We state:

*Page #21, line 321-326 “Recognizing that as many children die following discharge as during the acute phase of illness, the impact of HAP on this phase of recovery has not previously been explored, and regardless of the typical limitations to HAP exposure measurement that may exist in our analysis, these results must spur further investigations into the increasingly topical and relevant area of the post-acute illness period. It is this distinction which makes our study unique and relevant.”*

We hope that this properly contextualizes our results.

**Reviewer #2 Comment:**

**The future studies should use objective measures like indoor air sensors for PM2.5 to strengthen causality claims. Only 6 hospitals were included, even though they serve a large area. So, acknowledging the regional diversity within Uganda; broader national sampling could be used for claiming representativeness. Although interaction terms were tested, deeper analysis (e.g., stratified results by nutritional status) could reveal subgroup vulnerabilities' 6-month follow-up may not capture chronic effects of HAP. The longer-term studies are needed to observe delayed health impacts. While results are inconclusive, the study could better link findings to policy implications, like clean fuel promotion or post-discharge care programs. More discussion could be included on how cooking practices are culturally rooted, and how interventions should be community-sensitive.**

Thank you for this comment. It is extremely important to conduct a deeper analysis into how subgroup vulnerabilities may impact exposure and outcome. For this reason, we stratified our analysis by restricting the analysis to those with weight-for-age z-scores <-2 and found similar results. We have included the following in the results section:

*Page #17, lines 241-242 “We also stratified our analysis by anthropometry, restricting the analysis to those with weight-for-age z-scores <-2, and found similar results.”*

Furthermore, we agree that it is important to clearly outline how future studies can build on our work and put this into action items. To add additional clarity, we have added another sentence to the limitations which states that:

*Page #21, line 313-315 “Future studies should incorporate more objective measures of HAP, such as devices which directly measure PM2.5 exposure, to more accurately measure the relationship between HAP and post-discharge outcomes.*

With regards to promotion of clean fuel promotion in post-discharge care programs or other policy implications, we do not feel that these naturally flow from the results of this analysis. While we agree that such programs have ample justification from other research, linking them to this study seems somewhat problematic given our results. However, we do emphasize in our discussion the need for more work, especially related to longer term outcomes and more accurate measures of exposure measurement.
