# Supplementary material for: Exposure to pollutants for household cooking and lighting and pediatric post-discharge mortality following a severe infection in Uganda
Source: PLoS One. 2025 Jul 9;20(7):e0326105. doi: 10.1371/journal.pone.0326105 (PMC12240310; doi:10.1371/journal.pone.0326105)
Supplement: Attachment — Submitted filename: Second reviewer comments.docx [file pone.0326105.s007.docx]

Reviewer comments:

**Manuscript**

**Exposure to pollutants for household cooking and lighting and pediatric post-discharge mortality following a severe infection in Uganda**

1. Authors have adequately addressed the comments provided by the previous reviewer’s comments.
2. The revised version of the manuscript is much improved
3. Despite revision, considering the results of the study, further discussion of contextual factors is recommended to justify and make a useful study to improve method and tools for future study. Discussion on the limitation alone is not sufficed for future study in similar location or background.
